# Probing subwavelength in-plane anisotropy with antenna-assisted infrared nano-spectroscopy

**DOI:** 10.1038/s41467-021-22844-3

**Published:** 2021-05-11

**Authors:** Ziheng Yao, Xinzhong Chen, Lukas Wehmeier, Suheng Xu, Yinming Shao, Zimeng Zeng, Fanwei Liu, Alexander S. Mcleod, Stephanie N. Gilbert Corder, Makoto Tsuneto, Wu Shi, Zihang Wang, Wenjun Zheng, Hans A. Bechtel, G. L. Carr, Michael C. Martin, Alex Zettl, D. N. Basov, Xi Chen, Lukas M. Eng, Susanne C. Kehr, Mengkun Liu

**Affiliations:** 1 Department of Physics and Astronomy, Stony Brook University, Stony Brook, NY USA; 2 Advanced Light Source Division, Lawrence Berkeley National Laboratory, Berkeley, CA USA; 3 Institute of Applied Physics, Technische Universität Dresden, Dresden, Germany; 4 ct.qmat, Dresden-Würzburg Cluster of Excellence-EXC 2147, Technische Universität Dresden, Dresden, Germany; 5 Department of Physics, Columbia University, New York, NY USA; 6 State Key Laboratory of Low-Dimensional Quantum Physics, Department of Physics, Tsinghua University, Beijing, China; 7 Materials Sciences Division, Lawrence Berkeley National Laboratory, Berkeley, CA USA; 8 Department of Physics, University of California, Berkeley, CA USA; 9 Institute of Nanoelectronic Devices and Quantum Computing, Fudan University, Shanghai, China; 10 National Synchrotron Light Source II, Brookhaven National Laboratory, Upton, NY USA

**Keywords:** Nanoscience and technology, Infrared spectroscopy, Optics and photonics, Infrared spectroscopy

## Abstract

Infrared nano-spectroscopy based on scattering-type scanning near-field optical microscopy (s-SNOM) is commonly employed to probe the vibrational fingerprints of materials at the nanometer length scale. However, due to the elongated and axisymmetric tip shank, s-SNOM is less sensitive to the in-plane sample anisotropy in general. In this article, we report an easy-to-implement method to probe the in-plane dielectric responses of materials with the assistance of a metallic disk micro-antenna. As a proof-of-concept demonstration, we investigate here the in-plane phonon responses of two prototypical samples, i.e. in (100) sapphire and x-cut lithium niobate (LiNbO_3_). In particular, the sapphire in-plane vibrations between 350 cm^−1^ to 800 cm^−1^ that correspond to LO phonon modes along the crystal *b*- and *c*-axis are determined with a spatial resolution of < *λ*/10, without needing any fitting parameters. In LiNbO_3_, we identify the in-plane orientation of its optical axis via the phonon modes, demonstrating that our method can be applied without prior knowledge of the crystal orientation. Our method can be elegantly adapted to retrieve the in-plane anisotropic response of a broad range of materials, i.e. subwavelength microcrystals, van-der-Waals materials, or topological insulators.

## Abstract (web summary)

s-SNOM is a powerful tool, but it is less sensitive to in-plane variations. Here the authors present a method to improve this with a metallic microdisk antenna, which they demonstrate by probing in-plane phonon responses.

## Introduction

Scattering-type scanning near-field microscopy (s-SNOM) has emerged as a capable optical characterization tool^1,2^ and been widely used for probing nanoscale quantum phenomena^3–9^. In the recent decade, s-SNOM has demonstrated phase-resolved nano-spectroscopy at extreme sub-wavelength scales^10–12^, which has been applied for the identification of local vibrational fingerprints of various materials^13–18^, quantitative determinations of the local carrier density in semiconductors^19–22^, and investigations of surface polariton dispersions^23–26^. Light sources suitable for s-SNOM include broadband femtosecond lasers^27,28^, tunable gaseous or quantum cascade lasers^29^, thermal radiation sources^30–32^, free-electron lasers^33–35^, and synchrotron light^36,37^. Since its introduction, s-SNOM has been demonstrated to be less sensitive to the in-plane sample anisotropic response. This is primarily because the axisymmetric and elongated geometry of the atomic force microscope (AFM) tip shank leads to larger out-of-plane polarizability^38,39^. In uniaxial materials, the contributions from in-plane and out-of-plane components are described by several models^40–42^. Dielectric constants along these directions can be extracted with the help of waveguide modes^43^ or dispersive substrate^44^. Meanwhile, the near-field contrast arising from in-plane anisotropy has been predicted to be small for standard s-SNOM measurements^45^. This shortcoming not only impedes the nanoscale investigation of in-plane sample responses but also limits the anisotropic sensitivity of the near-field technique, making it, in certain cases, less desirable compared to its far-field counterparts such as ellipsometry. Enhanced in-plane near-field contrast has been realized with the second harmonic generation induced by a tilted tip^46^, or by probing at the proximity of strong phonon resonance frequencies^34,47–51^. However, these methods either require alteration of the conventional s-SNOM setup, or prior knowledge of the materials (phonon frequencies, non-zero second-order optical susceptibility, etc.), which implies difficulties for investigating new materials at a broad spectral range.

On the other hand, optical antennas are widely used in photonic devices^52^. An antenna can convert propagating light into the spatially confined evanescent field with specific in-plane momentum or polarization^53^. Optical antenna resonances have been extensively studied using s-SNOM^54–56^. Moreover, the concept of antenna-enhanced spectroscopy has existed for years^57–60^. It has also been demonstrated that antennas can enhance the contrast and sensitivity of near-field nano-spectroscopy via locally boosting the field intensity^61–63^. However, using antennas to selectively enhance the directional (in-plane) response of s-SNOM has not been theoretically nor experimentally demonstrated. Because of the in-plane dipolar geometry of most optical antennas, it primarily couples to the in-plane permittivity of the substrate, complementary to the typical measurement scheme of s-SNOM.

In this work, we demonstrate an easy-to-implement method for nano-spectroscopy with enhanced in-plane sensitivity, especially in-plane phonon responses, by making use of an off-resonant plasmonic disk antenna. The antenna functions as a messenger that converts the in-plane substrate dielectric response to an out-of-plane field intensity and phase contrast, which can be readily measured by s-SNOM nano-spectroscopy.

## Results

### Experimental schematic

As a proof-of-concept demonstration, we use s-SNOM to investigate the anisotropic in-plane phonon responses of sapphire (*α*-Al_2_O_3_), an extensively used substrate material for thin-film growth. This is achieved by measuring the angle-dependent plasmonic responses of a micro-sized gold disk antenna on the sapphire substrate. The shape of the antenna is chosen such that the structure remains invariant under sample rotation, which eliminates variances induced by geometric factors. The plasmon in the confined metal structure displays strong (“bright spot”) and weak (“dark spot”) field confinement at the tip–sample interface, which originates from the tip–disk interaction that captures the relative phase difference between the dipole moments of the tip and the disk^64–67^ (see the section “Discussion” and Supplementary Note 3 for further details). Comparing the spectra of the “bright spot” to the “dark spot” provides insight into the in-plane dielectric properties of the substrate. The advantage is that our method can, without any model fitting, determine the orientation-dependent transverse optical (TO) and longitudinal optical (LO) phonon frequencies of the substrate with a spectral accuracy down to 10 and 3 cm^−1^, limited by noise and spectral resolution, respectively. The downside of our method is that it requires fabricating antennas on the sample surface and sacrifices the highest achievable spatial resolution of conventional broadband s-SNOM, typically ~20 nm. In the mid-IR frequency range (8–25 μm), the spatial resolution of our method is determined by the diameter of the disk, which is demonstrated down to 1 μm in this work.

**Fig. 1:**
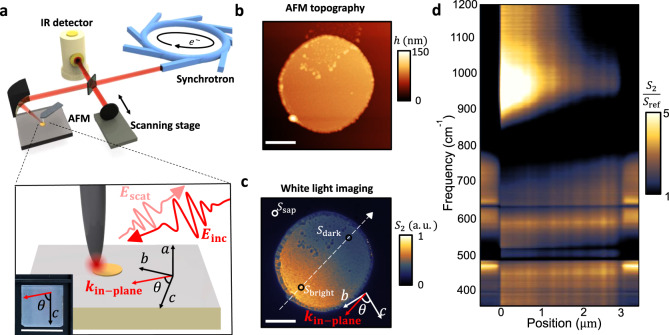
Experimental setup and bright–dark near-field contrast in gold disk on (100) sapphire. **a** Schematics for the s-SNOM nano-spectroscopy setup and sample crystal orientation. Inset at bottom shows the photo of the sapphire wafer. The *c*-axis is identified by corner-cuts. The scale bar is 10 mm. **b** AFM topography image of the disk antenna on (100) sapphire. **c** The corresponding white light near-field image. *k*_in-plane_ denotes the in-plane component of the incident light wavevector. Scale bars are 1 µm in both **b** and **c**. **d** Hyperspectral image normalized to a separate gold reference $\frac{{S}_{2}(\omega ,x)}{{S}_{{\rm{ref}}}(\omega )}$ obtained by scanning the tip across the disk (indicated by the white dashed arrow in **c**) while collecting a spectrum at each pixel. Here ${\theta }\approx {9}{{0}}^{\circ }.$ A gaussian filter of small width is imposed on the hyperspectral image for reducing noise and better display without creating distortion.

The top panel of Fig. 1a depicts the nano-spectroscopy (nano-FTIR) experimental setup in which synchrotron broadband light (Advanced Light Source at Lawrence Berkeley National Laboratory, Beamline 2.4) is coupled into a commercial s-SNOM system (Neaspec NeaSNOM) consisting of an asymmetric Michelson interferometer and an AFM. In one arm of the interferometer, the synchrotron light is focused on the apex of an AFM tip, which vibrates at its mechanical resonance frequency Ω and modulates the near-field interaction. The other arm of the interferometer consists of a moving mirror that alters the pathlength difference between the two optical paths. Demodulating the detected interference signal at higher harmonics of the tip vibration frequency (*n*Ω with *n* ≥ 2) suppresses the undesired far-field background and results in a local near-field response with a spatial resolution of ~20 nm, similar to the tip apex radius. Fourier transformation of the demodulated signal provides both amplitude and phase sensitivity due to the asymmetric Michelson interferometer setup.

Sapphire is known to exhibit strong uniaxial anisotropy in the infrared (IR) spectral range where the permittivity along the crystal *c*-axis is distinct from that along *a*- or *b*-axis (${\varepsilon }_{a}={\varepsilon }_{b}\ne {\varepsilon }_{c}$)^68^. We select (100) (m-cut) sapphire crystal as our proof-of-principle sample. A gold disk antenna of 3 μm diameter and 60 nm thickness is fabricated on the surface using standard e-beam lithography methods. The crystal orientation is schematically shown in the bottom panel of Fig. 1a. The oblique incidence of the p-polarized light leads to an in-plane wavevector component *k*_in-plane_. The angle between *k*_in-plane_ and the crystal *c*-axis is labeled as *θ*, which can be gradually varied in our experiments by rotating the sample. The AFM topography of the disk antenna is shown in Fig. 1b and the spectrally integrated (“white light”) near-field amplitude image is shown in Fig. 1c. For mid-IR frequency regime, demodulation at the second harmonic 2Ω (*S*_2_) is usually sufficient to suppress the far-field background. Therefore, without specification, the near-field spectra in the rest of the article are demodulated at the second harmonic.

### Bright-dark near-field contrast in micro disk antenna

The position-dependent signal intensity in the near-field image is due to the plasmonic response of the antenna and its interaction with the substrate and the tip^63–65,69,70^. As will be shown later, at each crystal orientation (different *θ*) we collect near-field amplitude and phase spectra at three important locations circled in Fig. 1c, labeled as ${S}_{{\rm{bright}}}(\omega ,\theta )$, ${\phi }_{{\rm{bright}}}(\omega ,\theta )$, ${S}_{{\rm{dark}}}(\omega ,\theta )$, ${\phi }_{{\rm{dark}}}(\omega ,\theta )$, and ${S}_{{\rm{sap}}}(\omega ,\theta )$, ${\phi }_{{\rm{sap}}}(\omega ,\theta )$, respectively. These spots are chosen because they best represent the properties of the plasmonic disk and its sapphire substrate. Near-field spectra on a separate bulk gold sample are also collected for reference and are denoted as ${S}_{{\rm{ref}}}(\omega )$ and ${\phi }_{{\rm{ref}}}(\omega )$. In Fig. 1d we show the hyperspectral image ($\frac{{S}_{2}(\omega ,x)}{{S}_{{\rm{ref}}}(\omega )}$) at $\theta \approx {90}^{\circ }$ to illustrate the position-dependent spectral response on and off the gold disk ($x$ depicts the position of the tip). The spectra are obtained by a line scan parallel to ${k}_{{\rm{in}}-{\rm{plane}}}$ from the bright side to the dark side as indicated by the white dashed arrow in Fig. 1c. The positions “0” and “3” μm mark the physical boundaries of the disk. Noticeably, the spectra on sapphire show multiple strong resonances due to the IR-active phonons below ~800 cm^−1^, as will be discussed in detail later. On the gold disk, unique signatures linked to the sapphire phonons are observed below ~700 cm^−1^. Above ~850 cm^−1^, a strong resonance can be observed and attributed to the geometric resonance of the disk, which occurs when the disk size matches roughly one-quarter of the wavelength. From Fig. 1d, we find that the “bright” and “dark” are relative terms specific to the frequencies. The assignment of “bright” and “dark” in the “white light” image shown in Fig. 1c is predominated by the geometric resonance of the gold disk at above ~850 cm^−1^.

### Low sensitivity to in-plane anisotropy from the bare sapphire substrate

**Fig. 2:**
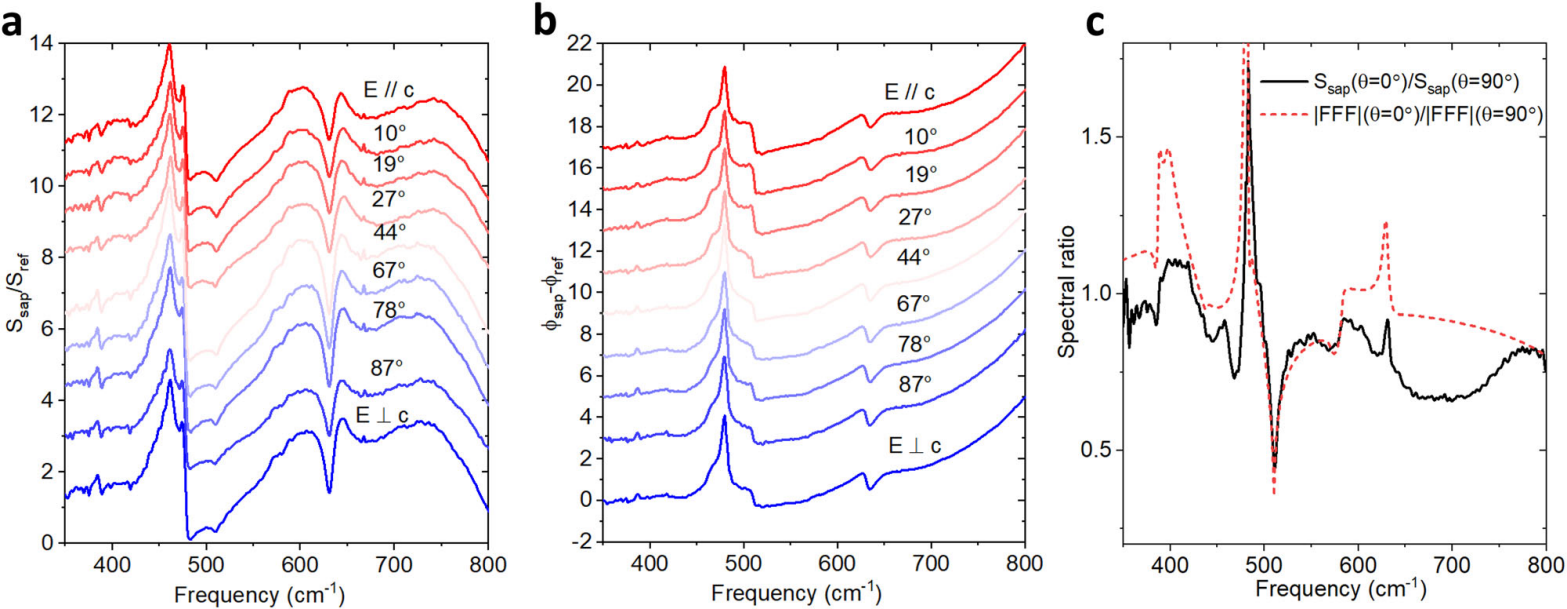
Sample orientation dependent near-field spectra on bare sapphire. **a** and **b** *θ*-dependent spectra of *S*_sap_/*S*_ref_ and *ϕ*_sap_−*ϕ*_ref_. Spectral feature (out-of-plane modes) changes at different angles are subtle. The spectra in **a** and **b** are vertically shifted for better display. **c** The ratio of *S*_sap_ between *θ* = 0° and 90° (black curve) plotted along with the ratio of FFF (far-field factor) at *θ*  = 0° and 90° (red dashed curve).

We first verify that the intrinsic near-field signal in a conventional s-SNOM configuration is less sensitive to the sample’s in-plane anisotropy. Figure 2a and b show $\theta$-dependent spectra on bare sapphire, with $\frac{{S}_{{\rm{sap}}}}{{S}_{{\rm{ref}}}}$ and ${\phi }_{{\rm{sap}}}-{\phi }_{{\rm{ref}}}$ at different $\theta$ angles from $E//c$ ($\theta =0^\circ$) to $E\perp c$ ($\theta =90^\circ$), where $E$ denotes the in-plane component of the electric field in the incident light. The incident light is p-polarized and thus the in-plane component of the electric field aligns to ${k}_{{\rm{in}}-{\rm{plane}}}$. Our experimental accuracy of $\theta$ is estimated to be $\pm 2^\circ$. Clearly, at different $\theta$ values, the changes are subtle, and the most prominent features are not directly related to the phonon frequencies. Moreover, since the spectra are taken on bare sapphire and then normalized to a separate gold reference sample, the multiplicative far-field factor (FFF) originating from secondary surface reflections is expected to modify the observed spectral response^29,71^. This $\theta$-dependent FFF is approximately ${\left(1+{r}_{p}\right)}^{2}$, where ${r}_{p}$ is the p-polarized Fresnel reflection coefficient. Figure 2c plots $\frac{{S}_{{\rm{sap}}}\left(\theta =0^\circ \right)}{{S}_{{\rm{sap}}}\left(\theta =90^\circ \right)}$ and $\frac{{\rm{|FFF|}}\left({\rm{\theta }}=0^\circ \right)}{{\rm{|FFF|}}\left({\rm{\theta }}=90^\circ \right)}$. The qualitative consistency shows that the slight difference observed in the $\theta$-dependent $\frac{{S}_{{\rm{sap}}}}{{S}_{{\rm{ref}}}}$ is largely due to the FFF instead of the intrinsic near-field response (see Supplementary Note 1 for further details).

### Amplitude ratio and phase difference in micro-disk antenna on sapphire

**Fig. 3:**
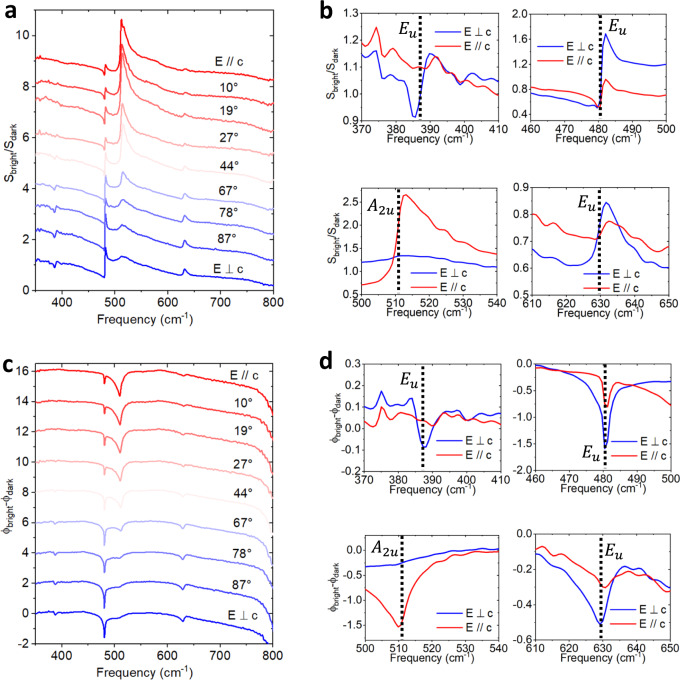
Anisotropic bright–dark contrast in a 3 μm Au disk antenna on sapphire. **a** ${\theta }$-dependent spectra of ${{S}}_{{\mathrm{{{bright}}}}}/{{S}}_{{\mathrm{{{dark}}}}}$. Spectral peaks emerge and disappear as ${\theta }$ rotates from ${0}^\circ$ to ${90}^\circ$. **b** A zoom-in comparison of ${{S}}_{{\mathrm{bright}}}/{{S}}_{{\mathrm{dark}}}$ at ${E}//{c}$ (red) and ${E}\perp {c}$ (blue) at different frequency ranges without vertical shift. **c** ${\theta }$-dependent spectra of ${{\phi }}_{{\mathrm{bright}}}-{{\phi }}_{{\mathrm{dark}}}$. **d** A zoom-in comparison of ${{\phi }}_{{\mathrm{bright}}}-{{\phi }}_{{\mathrm{dark}}}$ at ${E}//{c}$ and ${E}\perp {c}$ at different frequency ranges without vertical shift. The black dashed lines in **b** and **d** indicate the LO phonon frequencies^68^. For ${{E}}_{{u}}$ phonons (${E}\perp {c}$), ${{\omega }}_{{\mathrm{LO}}}={387.6},{481.7},$ and ${629}.{5}\,{\mathrm{cm}}^{-1}$. For ${{A}}_{{2u}}$ phonons (${E}//{c}$), ${{\omega }}_{{\mathrm{LO}}}={510.9}\,{\mathrm{cm}}^{-1}$. The spectra in **a** and **c** are vertically shifted for better display.

Figure 3a shows the amplitude ratio $\frac{{S}_{{\rm{bright}}}}{{S}_{{\rm{dark}}}}$ at different $\theta$ angles. In contrast to Fig. 2a, distinctive spectral contrast changes are observed: peaks at ~390, ~480, and ~630 cm^−1^ start to emerge as the in-plane electric field gradually aligns with the *b*-axis, while the peak at ~510 cm^−1^ diminishes. ${S}_{{\rm{bright}}}$ and ${S}_{{\rm{dark}}}$ are spectra taken at opposite ends of the antenna, which are ~2.5 μm apart. Such a distance is small compared to the free space wavelength (12.5–28.6 μm in this work). Thus, the FFF of two spectra can be regarded as the same, since the areas illuminated by incident light overlap almost completely in two measurements. The ratio of the spectra cancels the FFF and yields authentic near-field response. The significant peak formation/disappearance is a clear indicator of the anisotropic in-plane dielectric response, as will be detailed later. In Fig. 3b we plot the zoom-in views of $\frac{{S}_{{\rm{bright}}}}{{S}_{{\rm{dark}}}}$ at $E//c$ and $E\perp c$. Interestingly, all of the spectral peaks are associated with the LO phonon frequencies of sapphire^68^, which are indicated by the vertical black dashed lines. More specifically, ${E}_{u}$ phonons (*a-* and *b*-axis phonons) can be observed when $E\perp c$ and ${A}_{2u}$ phonons (*c*-axis phonons) manifest when $E//c$. Notice that there is some mixture of modes that we attribute to the imperfection of alignment angle and the out-of-plane (*a*-axis) contribution of the substrate.

Compared to the amplitude spectra, the phase spectra are even more intriguing. Significant phase contrast ${\phi }_{{\rm{bright}}}-{\phi }_{{\rm{dark}}}$ between different angles $\theta$ can be observed in Fig. 3c. In a zoom-in view (Fig. 3d), we find that the dips of ${\phi }_{{\rm{bright}}}-{\phi }_{{\rm{dark}}}$ are almost perfectly coincident with the LO phonon frequencies within a ±1 cm^−1^ range. This makes the phase contrast an even better indicator for the phonon frequencies than the peaks in the amplitude spectra, as will be explained later in the article and in Supplementary Note 2. Importantly, the phase contrast is also clearly observable on a 1 μm disk, ~20 times smaller than the wavelength (see Supplementary Note 8). However, we note that the signal-noise-ratios of spectra taken on smaller disks are worse than those on larger disks, due to weaker bright–dark contrast (see Supplementary Note 5) and weaker overall signals on smaller disks^72^.

## Discussion

**Fig. 4:**
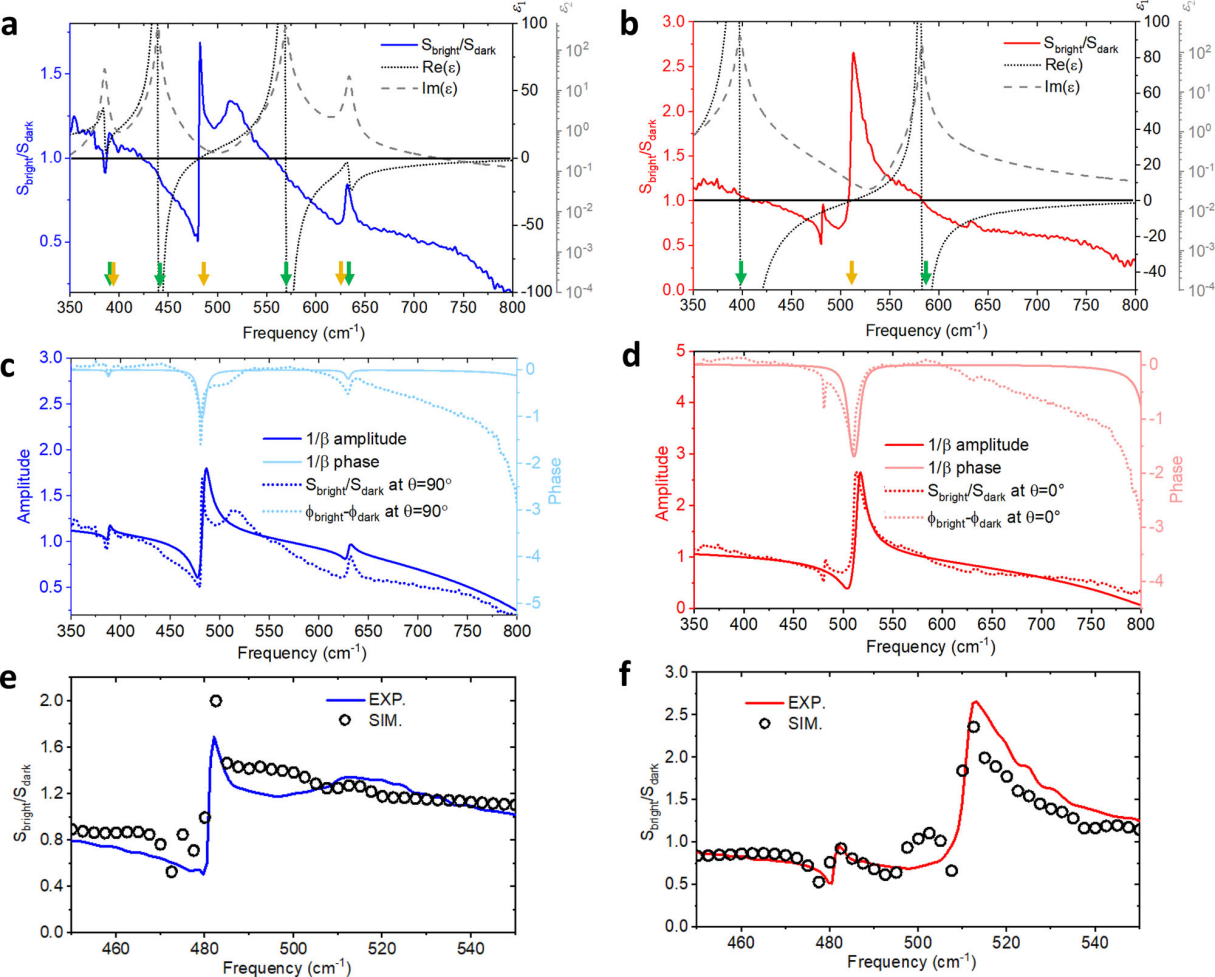
Analytical modeling and simulations. **a** $\frac{{{S}}_{{\mathrm{bright}}}}{{{S}}_{{\mathrm{dark}}}}$ at ${\theta } \sim {90}^\circ$ plotted along with the sapphire *b*-axis permittivity. **b** $\frac{{{S}}_{{\mathrm{bright}}}}{{{S}}_{{\mathrm{dark}}}}$ at ${\theta } \sim {0}^\circ$ plotted along with the sapphire *c*-axis permittivity. The green and yellow arrows in **a** and **b** indicate the ${{\omega }}_{{\mathrm{TO}}{i}}$ and ${{\omega }}_{{\mathrm{LO}}{i}}$, respectively. **c,** **d** amplitudes and phases of ${1}/{\beta }$ calculated with *b*-axis permittivity and *c*-axis permittivity together with experimental data. **e** and **f** simulated spectra plotted with experimental data for ${E}\perp {c}$ and ${E}//{c}$, respectively.

Next, we discuss the spectral features in the $\theta$-dependent $\frac{{S}_{{\rm{bright}}}}{{S}_{{\rm{dark}}}}$ spectra. It is instructive to plot $\frac{{S}_{{\rm{bright}}}}{{S}_{{\rm{dark}}}}$ at $\theta =90^\circ$ ($\theta =0^\circ$) together with the *b*-axis (*c*-axis) sapphire permittivity ${\varepsilon }_{b}$ $({\varepsilon }_{c})$, as shown in Fig. 4a and b). The permittivity of sapphire can be calculated by the conventional Lorentz oscillator function, which is commonly written in partial fraction decomposition form to explicitly indicate LO and TO phonon frequencies as

1 $$\varepsilon = \varepsilon_{\infty} \mathop{\prod}\limits_{i} \frac{{{\omega }_{{\rm{LO}}i}^{2}-{\omega }^{2}-i\omega {\gamma }_{{\rm{LO}}i}}}{{{\omega }_{{\rm{TO}}i}^{2}-{\omega }^{2}-i\omega {\gamma }_{{\rm{TO}}i}}},$$

where ${\omega }_{{\rm{TO}}i}$ and ${\omega }_{{\rm{LO}}i}$ are the *i*th TO and LO phonon frequencies. ${\gamma }_{{\rm{TO}}i}$ and ${\gamma }_{{\rm{LO}}i}$ are the corresponding damping coefficients. ${\varepsilon }_{\infty }$ is the high-frequency dielectric constant (parameters adapted from ref. ^68^). Three straightforward and important observations of the links between the $\frac{{S}_{{\rm{bright}}}}{{S}_{{\rm{dark}}}}$ spectra and the sapphire permittivity can be found: (1) the peaks in $\frac{{S}_{{\rm{bright}}}}{{S}_{{\rm{dark}}}}$ exhibit Fano-like line shape and correspond to $|\varepsilon | \sim 0$ (near LO phonon frequencies); (2) When $\varepsilon$ is large (near TO phonon frequencies), $\frac{{S}_{{\rm{bright}}}}{{S}_{{\rm{dark}}}} \sim$1; 3) Generally speaking $\frac{{S}_{{\rm{bright}}}}{{S}_{{\rm{dark}}}} > 1$ when ${\varepsilon }_{1}={Re}\left(\varepsilon \right) > 0$ and $\frac{{S}_{{\rm{bright}}}}{{S}_{{\rm{dark}}}} < 1$ when ${\varepsilon }_{1} = {Re} \left(\varepsilon \right)\, <\, 0$.

The observations can be understood intuitively by a straightforward antenna analysis^73^. When an antenna is placed at the interface of two homogenous media (i.e. air and sapphire), its response is altered by the dielectric environment. In particular, when the substrate’s permittivity is close to zero (epsilon-near-zero or ENZ), highly resonant behavior is triggered, despite the dimension and geometry of the antenna itself^70,74^. This resonance therefore emerges near LO phonon frequencies (marked by yellow arrows in Fig. 4a and b) and leads to strong local electric field enhancement. The high contrast between the “bright” and “dark” regions of this resonating mode can be picked up by near-field measurements. Near the TO phonon frequencies (marked by green arrows in Fig. 4a and b), the permittivity – especially the imaginary part – is large (also known as the epsilon-near-pole or ENP) and the antenna response is strongly suppressed. Therefore, at ENP, the antenna shows a spatially homogenous near-field signal, where ${S}_{{\rm{bright}}}\approx {S}_{{\rm{dark}}}$ or $\frac{{S}_{{\rm{bright}}}}{{S}_{{\rm{dark}}}} \sim 1$.

For a semi-quantitative understanding, we treat the disk-sapphire system quasi-statically and find out that $\frac{{S}_{{\rm{bright}}}}{{S}_{{\rm{dark}}}}$ contrast agrees well with the effective in-plane polarizability ${\alpha }_{{\rm{eff}}}$ of the metal disk. Under static electric field ${E}_{{\rm{inc}}}$, the resulting disk dipole moment is $p={\alpha }_{{\rm{disk}}}\left({E}_{{\rm{inc}}}-\frac{{p}^{{\prime} }}{32\pi {d}^{3}}\right)$, where ${\alpha }_{{\rm{disk}}}$ is the bare in-plane polarizability of the disk, ${p}^{{\prime} }=-\beta p$ is the image dipole moment, $\beta =\frac{\varepsilon -1}{\varepsilon +1}$ is the quasi-static reflection coefficient, $\varepsilon$ is the substrate in-plane permittivity, and $d$ is the distance of the induced dipole above the substrate surface^75^, which is in the same order as antenna thickness. As a result, ${\alpha }_{{\rm{eff}}}=\frac{{\alpha }_{{\rm{disk}}}}{1-\frac{{\alpha }_{{\rm{disk}}}\beta }{32\pi {d}^{3}}}\,$. Since ${\alpha }_{{\rm{disk}}}$ scales with the volume of the disk, which is much larger than ${d}^{3}$, ${\alpha }_{{\rm{eff}}}\propto 1/\beta$. In Fig. 4c and d, we plot the amplitudes and phases of ${1/\beta }_{b-{\rm{axis}}}=\frac{{\varepsilon }_{b}+1}{{\varepsilon }_{b}-1}$ and ${1/\beta }_{c-{\rm{axis}}}=\frac{{\varepsilon }_{c}+1}{{\varepsilon }_{c}-1}$, respectively. Note that to better match the experimental peak width and height, the damping rates for the ${A}_{2u}$ LO phonon at 510.9 cm^−1^ and ${E}_{u}$ LO phonon at 481.7 cm^−1^ are artificially broadened from 1.1 and 1.9 cm^−1^ to 8.8 and 7.6 cm^−1^, respectively in the calculated permittivity. This broadening is justified due to the limited spectral resolution (3.3 cm^−1^) of the instrument and the extra damping caused by the tip–sample interaction^76,77^. The line shapes of both the amplitude and phase strongly resemble the experimental spectra. Particularly, the phase of $1/\beta$ peaks when $|\varepsilon | \sim 0$, making the phase a good indicator for LO phonon frequencies (see Supplementary Note 2 for further details). The discrepancy in phase at high frequencies is due to the intrinsic geometric resonance of the disk above 850 cm^−1^, where the disk is similar to a quarter-wavelength antenna. This effect is not accounted for in the $1/\beta$ model.

The reason we resolve a bright–dark near-field contrast that tracks the effective disk polarizability is due to the tip–disk interaction. The tip effectively resolves the charge density distribution at the two ends of the disk^67^ via the relative phase difference between the dipole moments of the tip and the disk. This can be proven with numerical simulations (see Supplementary Note 3 for details) following the recently proposed simulation scheme with realistic tip modeling and signal demodulation^78^. To capture the major features while saving computational power, we carry out 2D FEM simulations, where a 3-μm-long antenna is placed on a sapphire substrate. The AFM tip is modeled as a truncated triangle with 20 μm height, 5 μm top radius, and 25 nm bottom radius, and the light excitation is modeled as a plane wave incident at a 60° angle with respect to the surface normal, as in the experimental setup. The permittivity tensor of sapphire used above is also applied in the simulation. In Fig. 4e and f the experimental spectra and the simulated spectra are plotted together, highlighting consistencies between them. The simulations are restricted to a 450–550 cm^−1^ window due to time and computational power constraints, emphasizing the *E*_*u*_ LO phonon at 481.7 cm^−1^ and the *A*_*2u*_ LO phonon at 510.9 cm^−1^. Nonetheless, the phonon signatures within that window are clearly reproduced. We note that without including the tip in the simulation, a correct spectral shape cannot be obtained (see Supplementary Fig. 2 for details).

**Fig. 5:**
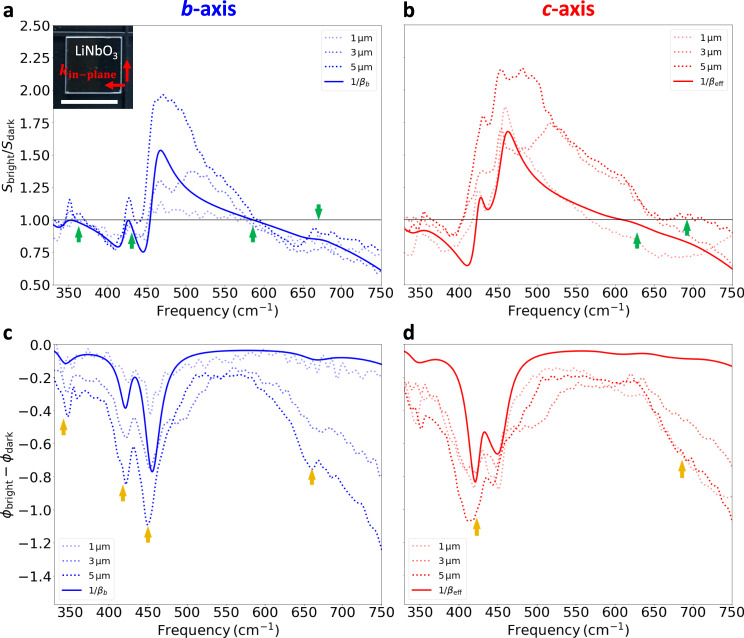
Anisotropic bright–dark contrast in Au disks with various diameters on LiNbO_3_. **a,** **b** ${{S}}_{{\mathrm{bright}}}/{{S}}_{{\mathrm{dark}}}$ at two orthogonal sample orientations for gold disks on LiNbO_3_, respectively. The green arrows in **a** and **b** indicate the ${{\omega }}_{{\mathrm{TO}}{i}}$ along *b*- and *c*-axes, respectively. The diameters of the disks are 1 μm, 3 μm, and 5 μm. $\left|{1}/{{\beta }}_{{b}}\right|$ is plotted in **a**. $|{1}/{{\beta }}_{{\mathrm{eff}}}|$ with ${{\varepsilon }}_{{\mathrm{eff}}}=\sqrt{{{\varepsilon }}_{{b}}{{\varepsilon }}_{{c}}}$ is plotted in **b**. Inset of **a** shows a photo of the LiNbO_3_ wafer. Note there are no corner cuts to help identify the *c*-axis. The electric field is set to be parallel to the edges of the LiNbO_3_. The scale bar is 10 mm. **c,** **d** ${{\phi }}_{{\mathrm{bright}}}-{{\phi }}_{{\mathrm{dark}}}$ for the same disks under the same sample orientation with **a** and **b**, respectively. Yellow arrows in **c** and **d** indicate the ${{\omega }}_{{\mathrm{LO}}{i}}$ along *b*- and *c*-axes, respectively. ${1}/{{\beta }}_{{b}}$ and ${1}/{{\beta }}_{{\mathrm{eff}}}$ phases are shown in solid curves in **c** and **d**, respectively.

To further demonstrate the broad applicability of our method, we applied our methodology also to an x-cut lithium niobate (LiNbO_3_) sample and determined its *c*-axis orientation. LiNbO_3_ has wide applications in electro-optics^79^, nonlinear optics^80^, fiber-optics^81^, and quantum information^82,83^. Like sapphire, LiNbO_3_ exhibits uniaxial anisotropy where the permittivity along the crystal *c*-axis is distinctively different as compared to the *a*- or *b*-axis^84^. The single-crystalline wafer is double-sided polished and has a size of 10 mm × 10 mm with a 1 mm thickness. The *c*-axis lies in the sample plane and is oriented parallel to one of the square edges. Since there are no corner cuts that would allow us to clearly assign the *c*-axis to one of the in-plane directions, [see the inset of Fig. 5a] this sample constitutes an excellent test to explore the full potential and benefits of the Au-disk method here. We hence fabricated 60 nm-thick gold disks of various sizes onto that x-cut LiNbO_3_ sample surface and measured the bright/dark ratio with the incident electric field being oriented parallel to each side once, using the same method as for the sapphire case above. The method helped us determine the *c*-axis orientation.

Figure 5a–d show the amplitude ratio ${S}_{{\rm{bright}}}/{S}_{{\rm{dark}}}$ and phase difference ${\phi }_{{\rm{bright}}}-{\phi }_{{\rm{dark}}}$ of gold disks with diameters of 1, 3, and 5 μm on LiNbO_3_ with two orthogonal sample orientations. Anisotropy is observed for all disk sizes. We can infer the phonon frequencies of LiNbO_3_ along the two crystal orientations and hence determine its correct *c*-axis orientation by comparing to the literature^84^. The small peak between 400 and 450 cm^−1^ in Fig. 5a and the relative depths for dips centered at 418 and 450 cm^−1^ in Fig. 5c are well captured by the amplitude and phase of $\left|1/{\beta }_{b}\right|$, respectively. For all disks, ${S}_{{\rm{bright}}}/{S}_{{\rm{dark}}}$ are close to 1 at ${\omega }_{{\rm{TO}}}$, marked by green arrows in Fig. 5a. The dips of ${\phi }_{{\rm{bright}}}-{\phi }_{{\rm{dark}}}$ coincide with ${\omega }_{{\rm{LO}}}$, as indicated by yellow arrows in Fig. 5c. These similarities with sapphire results show the repeatability and robustness of our method. For the case of *E//c*, $1/{\beta }_{{\rm{eff}}}=\frac{{\varepsilon }_{{\rm{eff}}}+1}{{\varepsilon }_{{\rm{eff}}}-1}$ with ${\varepsilon }_{{\rm{eff}}}=\sqrt{{\varepsilon }_{a}{\varepsilon }_{c}}$ is applied to incorporate the out-of-plane (*a*-axis) contribution^34^. Due to higher intrinsic phonon damping of LiNbO_3_ than sapphire, the out-of plane contribution is more prominent, and the fitting quality is slightly worse. The change of contrast on different gold disk diameters can be attributed to finite-size effects of the disk, which may include finite in-plane scattering momentum and overlap of detected region. A detailed discussion will come in a future work.

In conclusion, the plasmonic disk antenna displays a modified resonance behavior due to the in-plane permittivity of the substrate. The antenna serves as a converter that transmits the in-plane interaction of the disk and substrate to the out-of-plane localized electric field. This is especially useful for identifying an epsilon-near-zero region, where a sharp resonance feature of the antenna can be clearly identified in real space. Therefore, LO phonon frequencies can be identified through nano-spectroscopy as a distinctive phase contrast. The methodology is particularly suitable for thick (e.g. > 10 μm) anisotropic crystals with moderate phonon resonance damping and wide separation between LO and TO phonons. Anisotropy can be easily identified by rotating the crystal axis of the sample, which is not obvious with a tip-on-bare-sample scheme. Our work is a firm step towards obtaining the full anisotropic dielectric tensor at the nanoscale. However, one important obstacle seems to lie in the fact that the electromagnetic damping by the tip or the disk cannot be quantified appropriately at this stage, which certainly calls for future investigation.

With an improved understanding and engineering of the antenna structures, we foresee interesting future studies on the anisotropic in-plane responses of a broad range of materials, especially samples with subwavelength size such as topological microcrystals. Metallic structures of different shapes and sizes can also be explored for significantly enhancing the technique sensitivity by utilizing the intrinsic geometric resonances of the antenna. Plasmonic materials or chiral molecules beyond metallic antennas could also be used to yield a finer spatial resolution or sensitivity of the photon chirality. For example, a gateable graphene disk or dipolar molecules can probably further improve the spatial resolution to about 100 nm, which is on the order of the wavelength of graphene plasmons^85^.

## Methods

### Sample preparation

Both m-cut (100) sapphire and x-cut LiNbO_3_ substrates are purchased from MTI Corp. The sapphire is 1-side-polished with 0.5 mm thickness, and the LiNbO_3_ is 2-side-polished with 1 mm thickness. Gold disks are fabricated on the polished surface by electron-beam lithography (EBL).

### Nano-FTIR

Nano-FTIR is performed with a commercial scattering-type near-field microscope (Neaspec GmbH, Germany) located at beamline 2.4, Advanced Light Source (ALS), Berkeley, USA. ALS provides broadband IR radiation of high illuminance. The AFM was operated in tapping mode with ~65 nm oscillation amplitude, with cantilever resonance frequency at ~250 kHz. The Cu:Ge detector is sensitive to low-frequency IR signal down to ~330 cm^−1^.

## Supplementary information

Supplementary Information

## Data Availability

The data that support the findings of this study are available from the corresponding author upon reasonable request.
