# Supplementary material for: Probing subwavelength in-plane anisotropy with antenna-assisted infrared nano-spectroscopy
Source: Nat Commun. 2021 May 11;12:2649. doi: 10.1038/s41467-021-22844-3 (PMC8113487; doi:10.1038/s41467-021-22844-3)
Supplement: Supplementary file 1 — Supplementary Information [file 41467_2021_22844_MOESM1_ESM.pdf]

Supplementary materials for

Probing subwavelength in-plane anisotropy with antenna-assisted infrared nano-spectroscopy

Ziheng Yao[1,2+], Xinzhong Chen[1+], Lukas Wehmeier[3,4], Suheng Xu[1,5], Yinming Shao[5], Zimeng Zeng[6], Fanwei Liu[6], Alexander S. Mcleod[5], Stephanie N. Gilbert Corder[2], Makoto Tsuneto[1], Wu Shi[7,8,9], Zihang Wang[8], Wenjun Zheng[1], Hans A. Bechtel[2], G. L. Carr[10], Michael C. Martin[2], Alex Zettl[7,8], D. N. Basov[5], Xi Chen[6], Lukas M. Eng[3,4], Susanne C. Kehr[3], Mengkun Liu[1,10*]

[+] *Contribute equally*

1. *Department of Physics and Astronomy, Stony Brook University, Stony Brook, NY, 11794, USA*
2. *Advanced Light Source Division, Lawrence Berkeley National Laboratory, Berkeley, CA 94720, USA*
3. *Institute of Applied Physics, Technische Universität Dresden, 01062 Dresden, Germany*
4. *ct.qmat, Dresden-Würzburg Cluster of Excellence-EXC 2147, Technische Universität Dresden, 01062 Dresden, German*
5. *Department of Physics, Columbia University, New York, NY, 10027, USA*
6. *State Key Laboratory of Low-Dimensional Quantum Physics, Department of Physics, Tsinghua University, Beijing, 100084, China*
7. *Materials Sciences Division, Lawrence Berkeley National Laboratory, Berkeley, CA, 94720, USA*
8. *Department of Physics, University of California, Berkeley, CA, 94720, USA*
9. *Institute of Nanoelectronic Devices and Quantum Computing, Fudan University, Shanghai, 200433, China*
10. *National Synchrotron Light Source II, Brookhaven National Laboratory, Upton, NY, 11973, USA*

*mengkun.liu@stonybrook.edu

Supplementary Note 1: Multiplicative Far-field Factor

The multiplicative far-field factor (FFF), which originates from the multiple reflections of the incident and scattered field off the sample surface, is known to be present in the near-field spectroscopy measurement if the spectrum is not normalized to a reference within the same illuminated area[1,2]. That is, a near-field amplitude spectrum measured on sapphire at θ then normalized to a separate gold sample can be written as

$$S_{\mathrm{sap}}(\omega,\theta) = \frac{|FFF_{\mathrm{sap}}|(\omega,\theta)s_{\mathrm{sap}}(\omega,\theta)}{|FFF_{\mathrm{Au}}|(\omega)s_{\mathrm{Au}}(\omega)},$$

where s_{sap} and s_{Au} are the pure near-field response on sapphire and gold.

Although the exact form of FFF is not well studied, $\left(1+r_p\right)^2$ has been demonstrated to be a good approximation[2], where r_p is the p-polarized Fresnel reflection coefficient. r_p takes the simple form

$$r_p = \frac{n_2\cos(\alpha_1) - n_1\cos(\alpha_2)}{n_2\cos(\alpha_1) + n_1\cos(\alpha_2)},$$

where n_1 and n_2 are the indices of refraction of environment and sample. In our case $n_1 = 1$ represents the air and n_2 represents the sapphire. α_1 and α_2 are the incident and transmission angles, which follow Snell's law: $n_1\sin(\alpha_1) = n_2\sin(\alpha_2)$. In the $E \perp c$ case, $n_2 = \sqrt{\varepsilon_a}$. In the $E//c$ case, $n_2 \approx \sqrt{\varepsilon_{\mathrm{eff}}}$ and $\varepsilon_{\mathrm{eff}} = \sqrt{\varepsilon_b\varepsilon_c}$.

To investigate the θ - dependence of s_{sap}, we calculate $\frac{S_{\mathrm{sap}}(\omega,\theta=0°)}{S_{\mathrm{sap}}(\omega,\theta=90°)} = \frac{|FFF_{\mathrm{sap}}(\omega,\theta=0°)|s_{\mathrm{sap}}(\omega,\theta=0°)}{|FFF_{\mathrm{sap}}(\omega,\theta=90°)|s_{\mathrm{sap}}(\omega,\theta=90°)}$. As shown in Fig. 2(c), $\frac{S_{\mathrm{sap}}(\omega,\theta=0°)}{S_{\mathrm{sap}}(\omega,\theta=90°)}$ and $\frac{|FFF_{\mathrm{sap}}|(\omega,\theta=0°)}{|FFF_{\mathrm{sap}}|(\omega,\theta=90°)}$ share very similar features. The peaks in $\frac{|FFF_{\mathrm{sap}}|(\omega,\theta=0°)}{|FFF_{\mathrm{sap}}|(\omega,\theta=90°)}$ correspond to phonon modes along the *c*-axis and dips correspond to phonon modes along the *b*-axis. Consequently, we shall have $\frac{s_{\mathrm{sap}}(\omega,\theta=0°)}{s_{\mathrm{sap}}(\omega,\theta=90°)} \approx 1$, i.e. θ-dependence of s_{sap} is very weak. Equivalently, the difference observed in $S_{\mathrm{sap}}(\omega,\theta)$ for different θ values is mainly from the far-field effect. Consequently, we postulate that if the experiment were done on a sapphire microcrystal, $S_{\mathrm{sap}}(\omega,\theta)$ would not vary with θ significantly.

Supplementary Note 2: Effective disk polarizability and $\frac{1}{\beta}$ behavior

As shown in the main text, the effective polarizability of the gold disk is approximately $\frac{1}{\beta}$, where $\beta = \frac{\varepsilon_{\mathrm{in-plane}}-1}{\varepsilon_{\mathrm{in-plane}}+1}$. Here we investigate the behavior of $\frac{1}{\beta} = \left|\frac{1}{\beta}\right| e^{i\varphi}$ around the epsilon-near-zero (ENZ) region closely. The amplitude of $\frac{1}{\beta}$ can be expressed as

$$\left|\frac{1}{\beta}\right| = \sqrt{\frac{(\varepsilon_1+1)^2+\varepsilon_2^2}{(\varepsilon_1-1)^2+\varepsilon_2^2}} = \sqrt{1+\frac{4\varepsilon_1}{(\varepsilon_1-1)^2+\varepsilon_2^2}},$$

where $\varepsilon_1 = Re(\varepsilon_{\mathrm{in-plane}})$ and $\varepsilon_2 = Im(\varepsilon_{\mathrm{in-plane}})$. While the phase is given by

$$\varphi = -\frac{\pi}{2} - \arctan\left(\frac{\varepsilon_1^2+\varepsilon_2^2-1}{2\varepsilon_2}\right).$$

In Supplementary Fig. 1(a), we show the $\left|\frac{1}{\beta}\right|$ as a function of ε_1 and ε_2. The global maximum occurs when $\varepsilon_1 = 1$ and $\varepsilon_2 = 0$. That is, the peaks in the $\frac{S_{\text{bright}}}{S_{\text{dark}}}$ spectra show up right next to the ENZ point as shown in Fig. 3(b), around $\varepsilon_1 = 1$. When $\varepsilon_1 = 0$, the amplitude of $\frac{1}{\beta}$ is constantly 1, which means the "bright" and "dark" sides changes place as $\varepsilon_1$switches sign. In Supplementary Fig. 1(b) the phase of $\frac{1}{\beta}$ is plotted. It's clear that non-zero phase occurs only around the ENZ region and peak exactly at $\varepsilon_1 = \varepsilon_2 = 0$, which explains why the phase spectrum $\phi_{\text{bright}} - \phi_{\text{dark}}$ in Fig. 3(d) is an excellent indicator of the LO phonon frequencies.

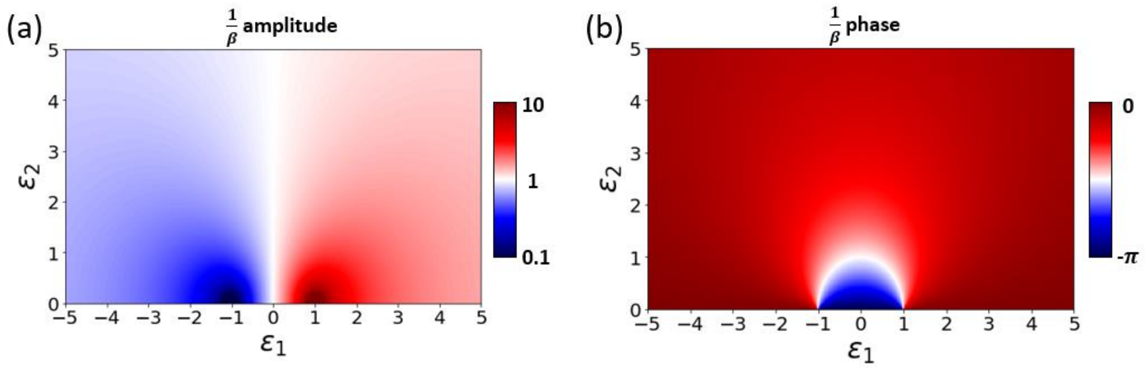

Supplementary Figure 1. Dependence of 1/*β* on *ε*. (a) Amplitude and (b) phase of $\frac{1}{\beta}$ as a function of $\varepsilon_1 = Re(\varepsilon_{\text{in-plane}})$ and $\varepsilon_2 = Im(\varepsilon_{\text{in-plane}})$ around the ENZ region.

Supplementary Note 3: Numerical simulations

Finite element method (FEM) simulations are performed using the RF module of the commercial platform COMSOL Multiphysics. The simulation approach is inspired by the recently proposed method for simulating the near-field interaction in s-SNOM[3]. Due to limited computational power, the simulations are performed in 2D rather than 3D. As schematically shown in Supplementary Fig. 2(a), the AFM tip is modeled as a truncated triangle with 20 μm height, 5 μm top radius, and 25 nm bottom radius. The light

excitation is modeled as a plane wave incident at a 60° angle with respect to the sample surface normal, as in the experimental setup. The simulation domain is set up as a circle surrounded by a thick layer of perfectly matched layers (PML). Compared to a rectangular domain typically used in numerical simulations, the circular domain matches the wavefront better, avoiding artifacts caused by sharp corners. The sample fills up the lower half of the simulation domain while the upper half contains the tip in an air environment.

Firstly, we demonstrate the validity of our 2D simulations. For such purpose, the electric field around the tip apex $E(x, y, h)$ is recorded while varying the tip-sample distance h, mimicking the vibration of the tip in the experiment. The tip motion is assumed to follow a harmonic function of time, i.e. $h(t) = A - A\cos(\Omega t) + h_0$, where $A = 25$ nm and $h_0 = 2$ nm, similar to realistic experimental values. $E(x, y, h)$ can be demodulated to the n-th harmonics of Ω by calculating the Fourier coefficient

$$E_n(x, y) = \frac{\Omega}{2\pi} \int_0^{2\pi/\Omega} E(x, y, h(t))\, e^{in\Omega t} dt.$$

The result for $n = 1, 2, 3, 4$ is shown in Supplementary Fig. 2(b). Compared to the full 3D case[3], the field patterns are the same except for some minor details. Thus, we deem the difference between 2D and 3D simulations is of secondary importance.

It has been demonstrated that the electric field near the tip apex is a quantitative gauge for the near-field interaction[3]. In the previous study, the volume-averaged field is considered. For simplicity, we show that the electric field taken at a single point (a few nm above the sample surface right under the tip apex) is already sufficient to capture the experimental observations. We verify this statement, together with the validity of our 2D simulations, by simulating the near-field spectrum of a SiO_2 sample. As shown in Supplementary Fig. 2(c), the third harmonic near-field spectrum on SiO_2 normalized to the spectrum on Si shows a signature phonon resonance around 1100 cm^{-1}, which is quantitatively consistent with previous experimental data and calculation[1,4]. For simulations shown in Fig. 4(e) and 4(f), an antenna of 3 µm length made of gold is added into the simulation and the sample is replaced by sapphire. Demodulated fields 500 nm away from both ends of the antenna are calculated then divided. The ratio is shown in Fig.

4(e) and (f) to compare with the experimental data. Due to computation power and time limitations, simulations are only carried out within the 450 cm^{-1} to 550 cm^{-1} window to capture the most prominent experimental features.

Another simulation using the finite-difference time-domain (FDTD) method (Lumerical FDTD) is carried out as a comparison. In this simulation, we exclude the tip and only consider the antenna on sapphire. The ratio of the electric field at two ends of the antenna are shown in Supplementary Fig. 2(d) and (e) for $E \perp c$ and $E//c$ cases, respectively. Without the tip, the spectral features also resemble the experimental observations, i.e. peaks occur near the LO phonon frequencies. However, the quantitative contrast value is significantly off compared to experimental data because the ratio is larger than 1 in most of the frequency range. Thus, our finding suggests that the tip plays an important role in the simulation of near-field interaction. The inclusion of the tip is mandatory for a quantitative simulation.

In fact, the appearance of bright and dark contrast on the micro antenna can be attributed to the tip-disk interaction[5–7], as shown in Supplementary Fig. 2(f). When illuminated by the incident light, the free carriers driven by the external electric field accumulate at the boundary of the disk, forming a (disk) dipole moment. Similarly, the metal-coated tip forms another dipole. The coupling strength (including both amplitude and phase) between the two dipoles varies as the tip moves from one end of the disk to the other, thus altering the scattering signal. In Supplementary Fig. 2(g), numerical simulations demonstrate this bright-dark contrast effect on various metallic structures with the presence of tip, similar to the observation in the main text.

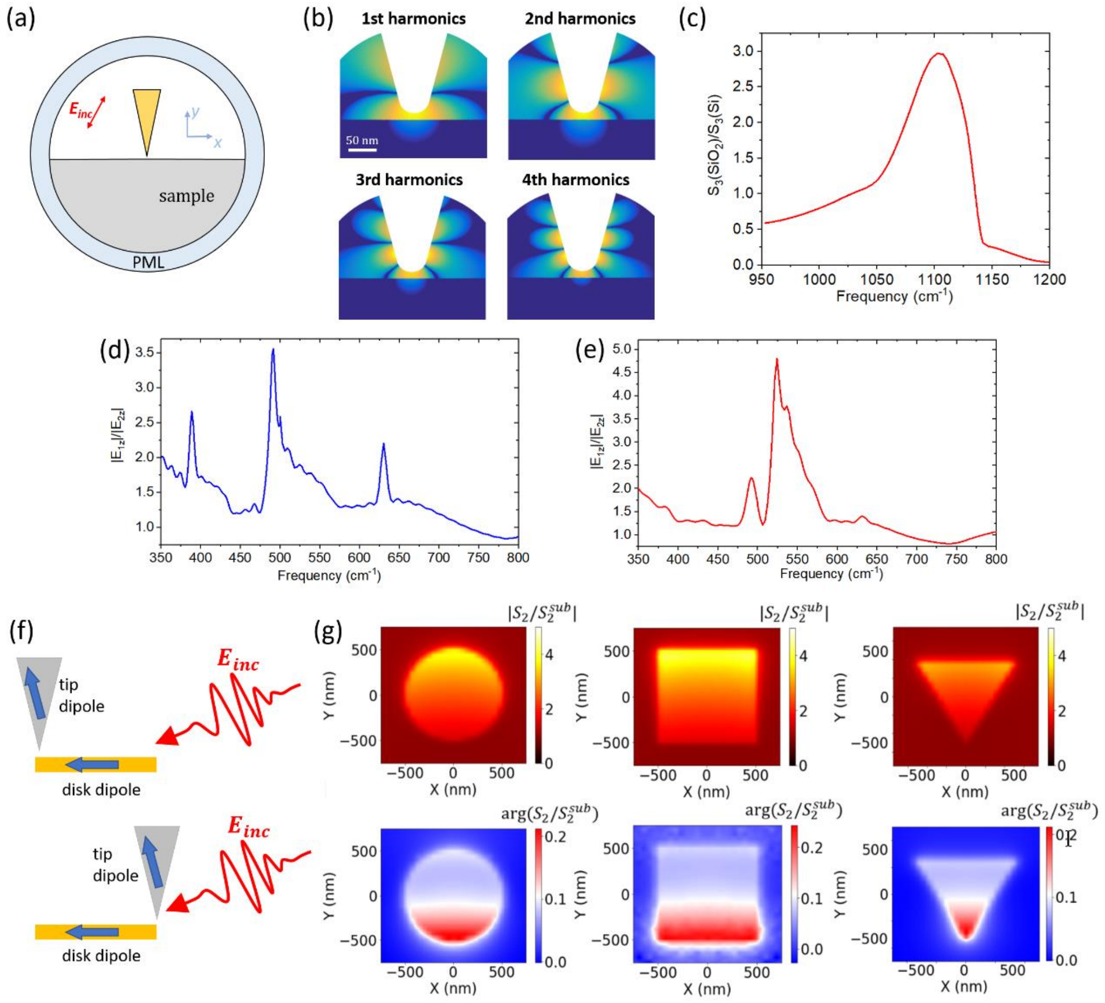

Supplementary Figure 2. Numerical simulations. (a) Schematics of FEM simulation. (b) Demodulated electric field distribution around the tip apex at different harmonics. (c) Simulated near-field spectrum on SiO_2 sample normalized to that on Si. (d) and (e) FDTD simulations of the field ratio above the disk without considering the presence of the tip for $E \perp c$ and $E // c$, respectively. (f) Schematics of different couplings between the tip dipole and the disk dipole as the tip moves from one end to the other. (g) Simulations of near-field scattering contrast on multiple metallic structures with the presence of tip. Light incidents from -*y* to +*y* direction with a 60 degree incident angle. The inhomogeneities in both amplitude (top row) and phase (bottom row) of the near-field signal are clearly present in all metal structures.

Next, we demonstrate through simulations that our proposed technique can be applied to measure the in-plane dielectric anisotropy of micro-crystals smaller than the wavelength. Assume the scenario where a 3 μm long antenna is fabricated on a sapphire

micro-crystal of size 10 μm and thickness of 1 μm. The sapphire micro-crystal is placed on a substrate with permittivity $\varepsilon = 2$ (Supplementary Fig. 3(a)). In Supplementary Fig. 3(b) we show the simulation results for two crystal orientations $E \perp c$ and $E//c$. Compared to results shown in Fig. 4(e) and (f), although the peaks are broadened and the contrast is reduced here, the main spectral features due to the in-plane anisotropic phonon response remain observable. This broadening is likely due to the influence of the substrate and the finite size of the micro-crystal.

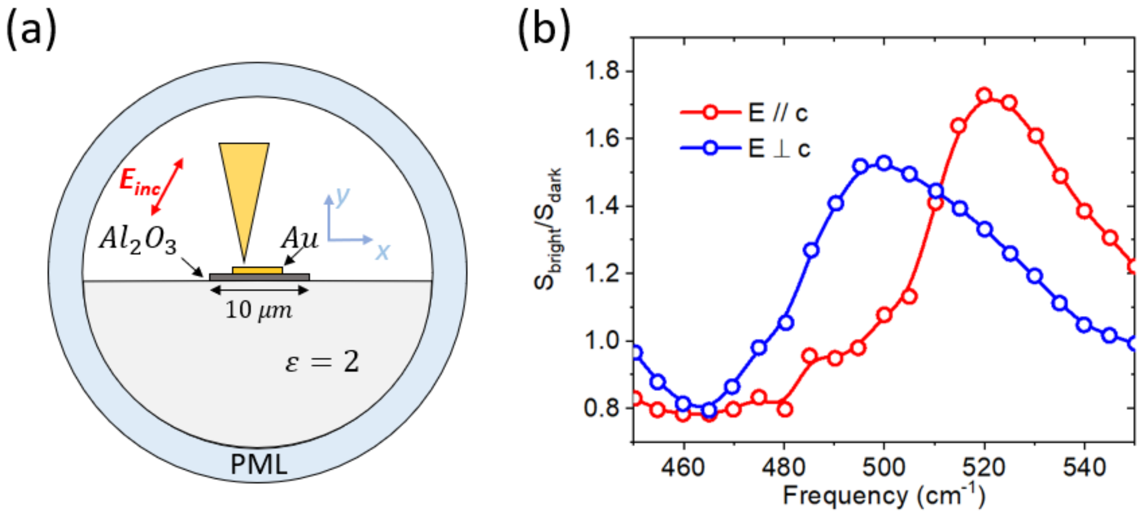

Supplementary Figure 3. FEM simulation on finite-size sapphire. (a) Schematics of the sapphire micro-crystal simulation. (b) simulated spectra for $E \perp c$ and $E//c$, respectively.

We also would like to comment on the effect of the Au disk antenna size. In the $1/\beta$ analysis above we demonstrate that the contrast of bright to dark flips at $\varepsilon = 0$ in the sample. In FEM simulations we monitor $\frac{S_{\text{bright}}}{S_{\text{dark}}}$ while changing the sample permittivity in the vicinity of ε =0. For a 3 μm antenna and 20 μm incident wavelength, $\frac{S_{\text{bright}}}{S_{\text{dark}}} = 1$ shows up at $\varepsilon \approx -0.7$. For a 1 μm antenna under the same illumination wavelength, $\frac{S_{\text{bright}}}{S_{\text{dark}}} = 1$ shows up at $\varepsilon \approx -0.4$. That is, the ratio of antenna size l and wavelength λ dictates the specific contrast. In the quasi-static limit $l/\lambda \to 0$, we expect it to recover exactly the $1/\beta$ behavior.

Supplementary Note 4: Sapphire film thickness dependence of the signal contrast

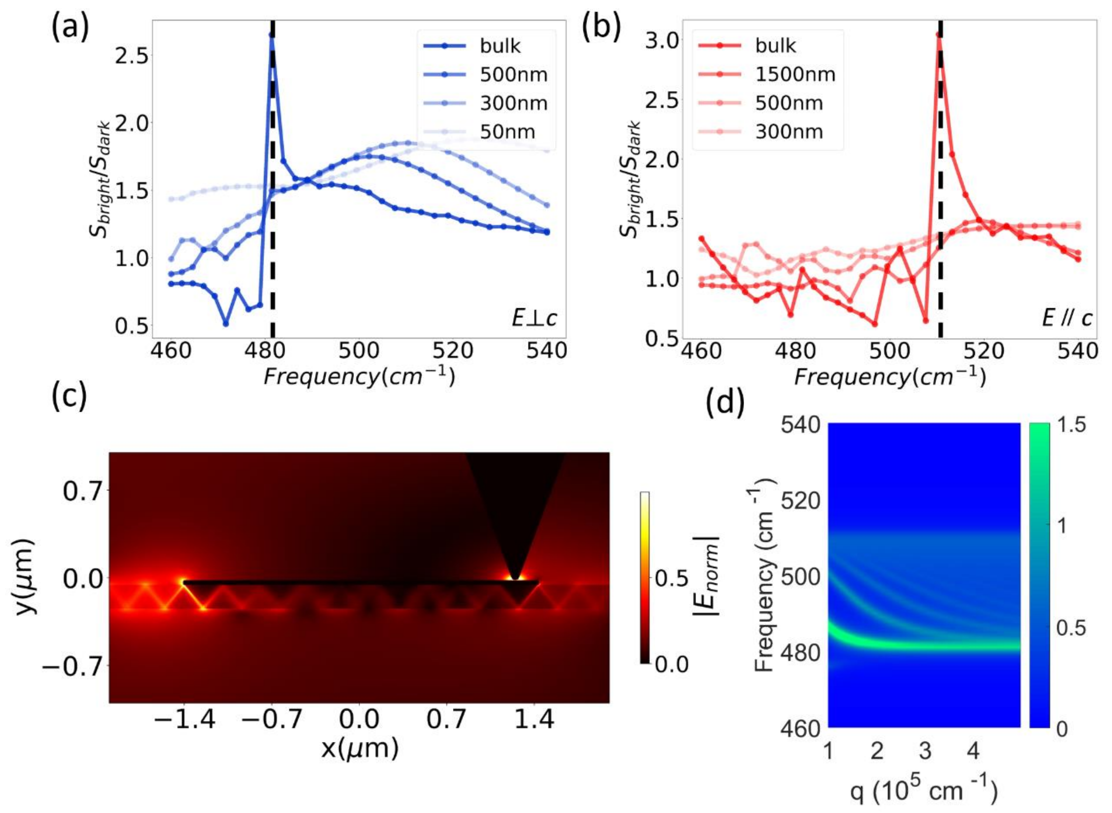

Supplementary Figure 4. Sapphire film thickness dependence of the signal contrast. (a) and (b) Simulated spectra of gold disk on sapphire thin films with different sapphire thicknesses for $\boldsymbol{E} \perp \boldsymbol{c}$ and $\boldsymbol{E}//\boldsymbol{c}$, respectively. The LO phonon frequencies at corresponding directions are shown in black dashed vertical lines. (c) Simulated electric field distribution for *E*//*c* with 300 nm sapphire thin films at 500 cm^{-1}. (d) Calculated dispersion for 300 nm sapphire thin films on Si substrate with *E*//*c*.

In Supplementary Fig. 4 (a) and 4(b), we show the sapphire film thickness-dependence of the $\frac{S_{\mathrm{bright}}}{S_{\mathrm{dark}}}$ signal contrast. For both crystal orientations, the peaks at LO phonon frequencies are damped when the film thickness decreases.The spectra for $E//c$ cases show relatively larger fluctuations than the $E \perp c$ cases and the peak height at the LO phonon resonance is more sensitive to the decrease of film thickness. It can be attributed to the effect of phonon polariton waveguide mode for *E//c* cases[8,9]. Supplementary Fig. 4(c) shows the simulated electric field distribution of the hyperbolic phonon polariton mode based on finite element modeling (FEM) simulation. Supplementary Fig. 4(d) shows the imaginary part of *p*-polarized Fresnel coefficient r_p, indicating the dispersion

relation of the phonon polariton in the sapphire-substrate system. The propagating waveguide mode interferes with the plasmonic response of the gold disk and our method is not appropriate for this situation. However, in this case, the optical anisotropy can be quantitatively extracted from the ordinary and extraordinary waveguide modes in thin film, which was realized in the previous study[10]. Our method, together with the waveguide modes analysis, establishes a general methodology for the optical anisotropy measurement on samples of great variety.

Supplementary Note 5: Disk diameter-dependence of the signal contrast

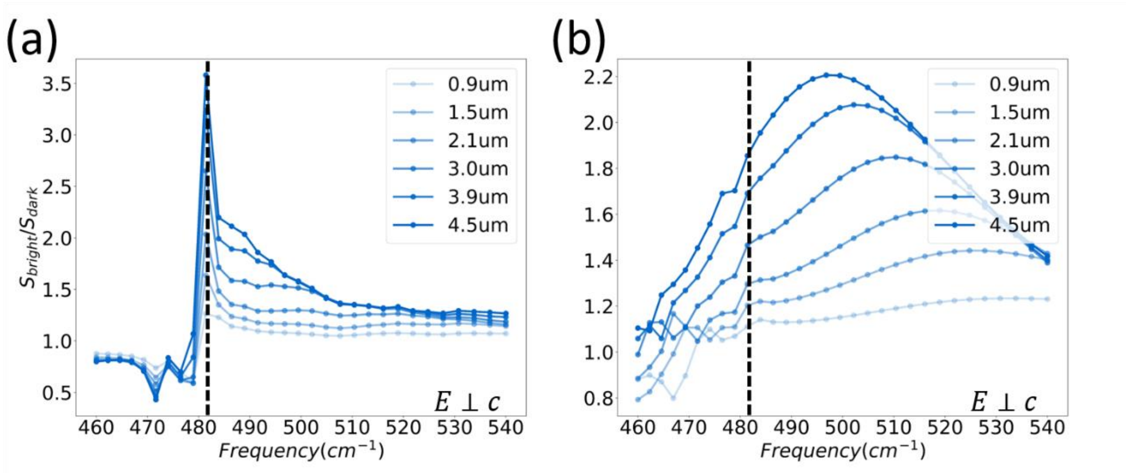

Supplementary Figure 5. Disk diameter-dependence of the signal contrast. (a) and (b) Simulated $\frac{S_{\text{bright}}}{S_{\text{dark}}}$ spectra of gold disk with different disk diameters on bulk sapphire (a) and 300 nm sapphire film (b), respectively. The electric field is perpendicular to *c*-axis. Black vertical dashed lines show the LO phonon frequency.

Next, we investigate the signal dependence on gold disk diameter. The overall value of the $\frac{S_{\text{bright}}}{S_{\text{dark}}}$ spectra increases monotonically with increasing disk diameter, indicates the positive correlation between the disk diameter and the effective polarizability of the system. The enhancement of plasmonic resonance can be utilized to magnify the resonance from the sample. In Supplementary Fig. 5 (a) and (b) we plot the simulated disk-diameter dependent $\frac{S_{\text{bright}}}{S_{\text{dark}}}$ spectra on bulk sapphire and on 300 nm sapphire film. The amplitude of the peak and signal contrast for 4.5 μm disk diameter is magnified compared with the one for 0.9 μm disk diameter.

Supplementary Note 6: Disk thickness-dependence of the signal contrast

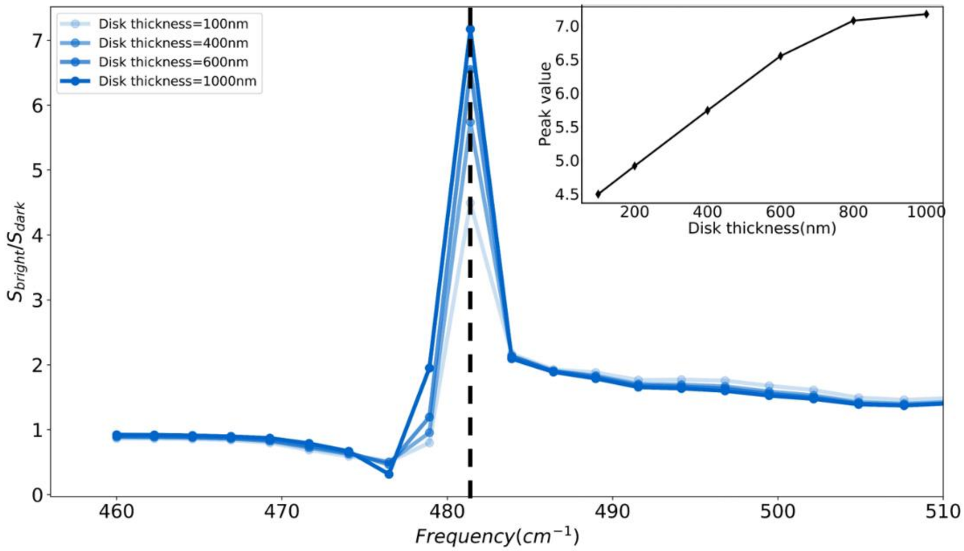

Supplementary Figure 6. Simulated $\frac{S_{\mathrm{bright}}}{S_{\mathrm{dark}}}$ spectra of gold disk on sapphire with different gold disk thicknesses with $E \perp c$. The inset shows the disk thickness dependence of amplitude of peak. The disk diameter is fixed at 3 μm. Black vertical dashed line shows the LO phonon frequency.

In Supplementary Fig. 6, we simulate the gold-disk thickness dependence of $\frac{S_{\mathrm{bright}}}{S_{\mathrm{dark}}}$ spectra for sapphire with $E \perp c$. The peak value increases almost linearly with increasing disk thickness until saturates around ~800 nm. The stronger signal can be attributed to the increase of gold disk volume, and therefore the increase of its polarizability. When the disk is too thick, the interaction between the disk plasmon and substrate gets weaker, so the contrast saturates.

Supplementary Note 7: Signal contrast for freestanding films

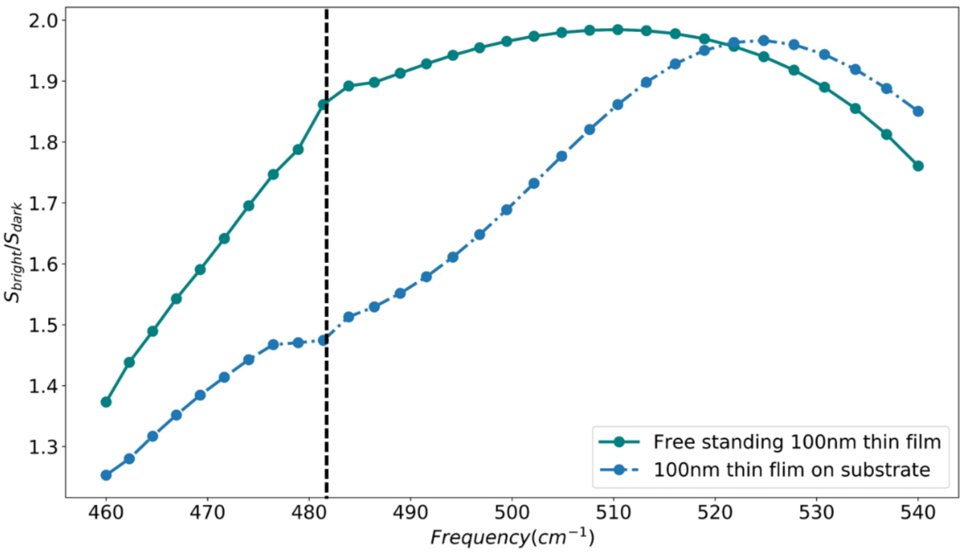

Supplementary Figure 7. Simulated spectra for freestanding 100 nm thin film of sapphire, and 100 nm thin film of sapphire on a substrate with dielectric constant 2+0.1i. Black vertical dashed line shows the LO phonon frequency.

To determine the contribution of the substrate when probing thin film samples, we simulate the $\frac{S_{\text{bright}}}{S_{\text{dark}}}$ on the freestanding thin film (100 nm sapphire) and thin film on a non-resonant substrate ($\varepsilon = 2 + 0.1i$). The contrast is stronger on a freestanding film, which may due to less "leakage" of the field into the substrate.

Supplementary Note 8: Spectral contrast on 1 μm disk

We also test our technique experimentally with a gold disk of 1 μm diameter. The $\frac{S_{\text{bright}}}{S_{\text{dark}}}$ and $\phi_{\text{bright}} - \phi_{\text{dark}}$ spectra for two orthogonal crystal orientations are shown in Supplementary Fig. 8. Although compared to results on the 3 μm disk the contrast is noticeably lower, the anisotropic response is clearly distinguishable. More specifically, in the phase spectrum $\phi_{\text{bright}} - \phi_{\text{dark}}$, the peaks still clearly indicate the LO phonon frequencies along two different crystal orientations. Therefore, we conclude that our method remains applicable to smaller antennas.

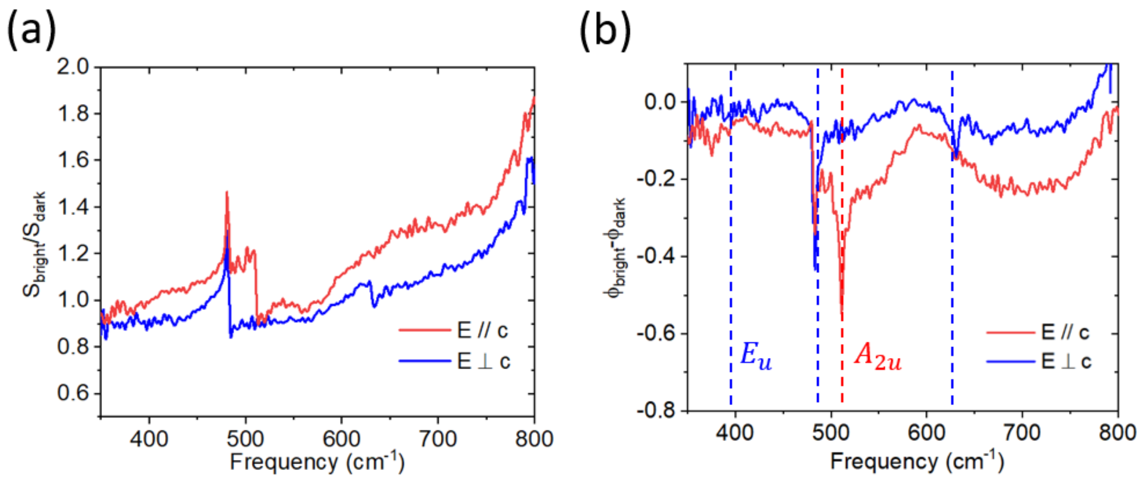

Supplementary Figure 8. On 1 μm disk on sapphire. (a) $S_{\mathbf{bright}}/S_{\mathbf{dark}}$ and (b) $\phi_{\mathbf{bright}} - \phi_{\mathbf{dark}}$ spectrum on 1 μm disk on sapphire for two orthogonal crystal orientations. Blue and red dashed lines in (b) indicate E_u and A_{2u} LO phonon frequencies respectively.

Supplementary Note 9: The spectrum of the synchrotron radiation on gold reference

In Supplementary Fig. 9 we show the near-field spectrum of the synchrotron radiation on a gold reference. In the 350 cm^{-1} to 800 cm^{-1} range, the sufficiently high radiation intensity guarantees a good signal-noise ratio. The lower frequency cutoff is due to the limited bandwidth of the IR detector we use.

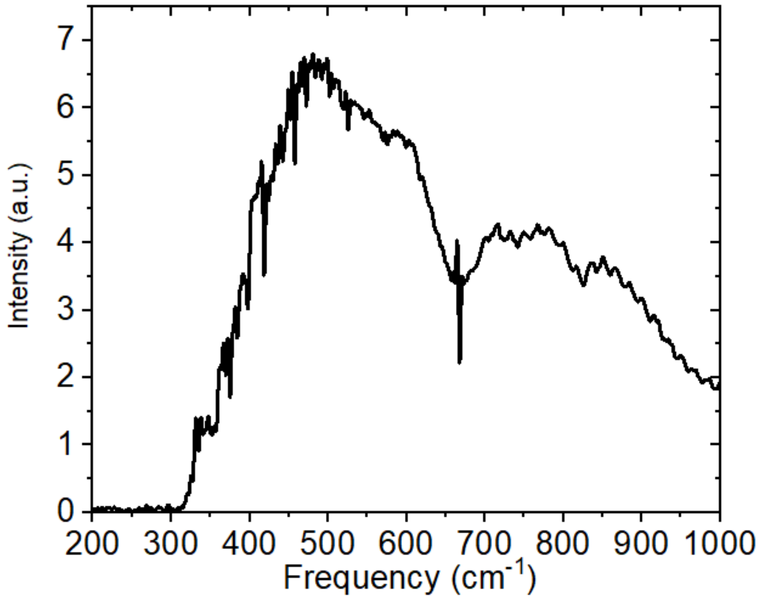

Supplementary Figure 9. Raw spectrum of the synchrotron radiation on gold reference.

Supplementary Note 10: The near-field phase of line scan across 3 μm antenna

In Supplementary Fig. 10 we show the hyperspectral near-field phase normalized to a separate gold reference sample obtained simultaneously with Fig. 1(d). 0 and 3 at *x*-axis denotes the boundary of the gold disk antenna. The E_u phonon frequencies around 480 cm^{-1} and 660 cm^{-1}, as well as the geometric resonance of the disk around 850 cm^{-1} can be observed.

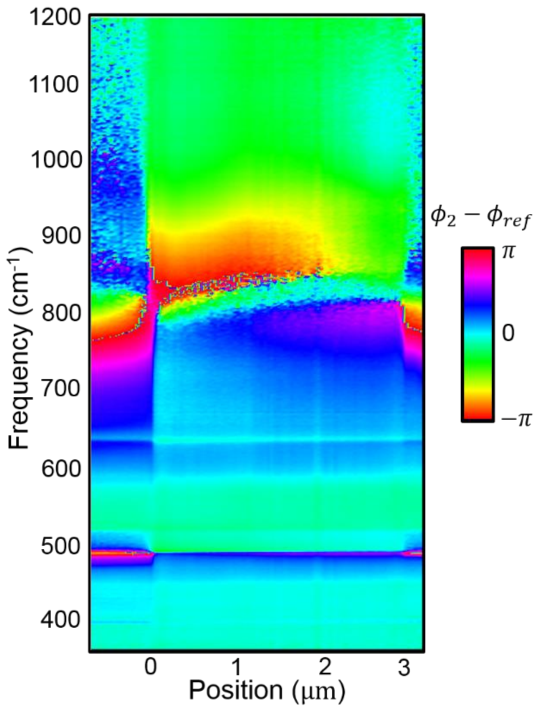

Supplementary Figure 10. Normalized near-field phase across the 3 μm gold disk antenna.

Supplementary References

1. Zhang, L. M. *et al.* Near-field spectroscopy of silicon dioxide thin films. *Phys. Rev. B* **85**, 075419 (2012).

2. Autore, M., Mester, L., Goikoetxea, M. & Hillenbrand, R. Substrate Matters: Surface-Polariton Enhanced Infrared Nanospectroscopy of Molecular Vibrations. *Nano Lett.* **19**, 8066–8073 (2019).

3. Mooshammer, F. *et al.* Quantifying Nanoscale Electromagnetic Fields in Near-Field Microscopy by Fourier Demodulation Analysis. *ACS Photonics* **7**, 344–351 (2020).

4. McLeod, A. S. *et al.* Model for quantitative tip-enhanced spectroscopy and the extraction of nanoscale-resolved optical constants. *Phys. Rev. B* **90**, 085136 (2014).

5. García-Etxarri, A., Romero, I., García de Abajo, F. J., Hillenbrand, R. & Aizpurua, J. Influence of the tip in near-field imaging of nanoparticle plasmonic modes: Weak and strong coupling regimes. *Phys. Rev. B* **79**, 125439 (2009).

6. Chen, X. *et al.* Rigorous numerical modeling of scattering-type scanning near-field optical microscopy and spectroscopy. *Appl. Phys. Lett.* **111**, 223110 (2017).

7. Walla, F. *et al.* Near-Field Observation of Guided-Mode Resonances on a Metasurface via Dielectric Nanosphere Excitation. *ACS Photonics* **5**, 4238–4243 (2018).

8. Dai, S. *et al.* Subdiffractional focusing and guiding of polaritonic rays in a natural hyperbolic material. *Nat. Commun.* **6**, 1–7 (2015).

9. Hu, G., Shen, J., Qiu, C. W., Alù, A. & Dai, S. Phonon Polaritons and Hyperbolic Response in van der Waals Materials. *Adv. Opt. Mater.* **8**, 1901393 (2020).

10. Hu, D. *et al.* Probing optical anisotropy of nanometer-thin van der waals microcrystals by near-field imaging. *Nat. Commun.* **8**, (2017).
